# Deciphering the potential of plant metabolites as insecticides against melon fly (*Zeugodacus cucurbitae*): Exposing control alternatives to assure food security

**DOI:** 10.1016/j.heliyon.2025.e42034

**Published:** 2025-01-16

**Authors:** Zinat Jahan Chowdhury, Anik Banik, Tanjin Barketullah Robin, Mohammed Rashed Chowdhury

**Affiliations:** a Department of Entomology, Sylhet Agricultural University, Sylhet, 3100, Bangladesh; b School of Environmental and Rural Science, University of New England, Armadale, NSW, 2351, Australia; c Department of Plant and Environmental Biotechnology, Sylhet Agricultural University, Sylhet, 3100, Bangladesh; d Faculty of Biotechnology and Genetic Engineering, Sylhet Agricultural University, Sylhet, 3100, Bangladesh; e Department of Biochemistry and Chemistry, Sylhet Agricultural University, Sylhet, 3100, Bangladesh

**Keywords:** Fruit fly, Molecular docking, Insecticide likeliness, Toxicity

## Abstract

In the absence of effective biological or chemical controls, the melon fly poses a significant threat to food security, particularly impacting cucurbit crops in tropical and subtropical regions. Melon fly infestations have resulted in yield losses of 30 %–100 %, depending on the specific cucurbit species and season. Current control methods using synthetic chemicals are challenging due to their environmental and biological impacts. This study identified 59 phytocompounds with potential insecticidal properties against the melon fly, exhibiting minimal environmental impact. Key protein targets—hedgehog protein, spastin protein, and ABC-type heme transporter ABCB6 protein—were selected for binding affinity analysis. Camptothecin demonstrated the highest binding affinities for hedgehog protein (−57.32 kcal/mol) and spastin protein (−50.84 kcal/mol), while jervine had the strongest binding affinity for ABC-type heme transporter ABCB6 protein (−43.92 kcal/mol). The control compound, malathion, showed lower binding affinities across all three proteins. Stability of the top compound-protein complexes was further confirmed through a 100 ns molecular dynamics simulation. In insecticide-likeness evaluations, jervine consistently scored high, with camptothecin also performing well, while neriifolin ranked lower. The leading compounds showed no adverse effects that could diminish their insecticidal potential. These findings indicate that jervine and camptothecin are promising candidates for melon fly management, offering potential to prevent significant crop losses. However, as this study was conducted solely through computational methods, we recommend subsequent *in vitro* and field trials for the future drug development.

## Graphical abstract

**Figure:**
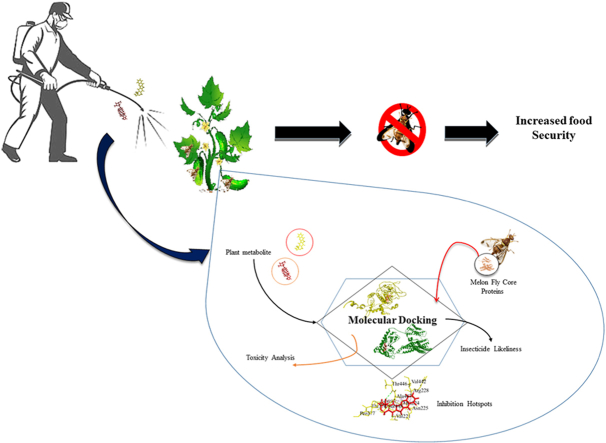

## 1 Introduction

Fruit flies (Family: Tephritidae, Diptera) are among the most devastating insect pests of fruits, vegetables, and ornamental plants worldwide [1]. This pest is considered a quarantine pest of most horticultural crops around the world [2]. The melon fly *Zeugodacus cucurbitae* (Coquillett), (formerly called *Bactrocera cucurbitae*) is an agricultural pest that attacks almost 16 kinds of cucurbits [3].Not only in cucurbits but this pest has also been recorded to attack tomatoes [4]. Adult female flies (*Z. cucurbitae*) puncture the soft fruits by inserting the ovipositor and lay eggs [5]. After hatching, the maggot creates a tunnel, feeds on the pulp and starts damage. As a result, the infested fruit may get rotten, dry out, shed early, occasionally develop deformities, and ultimately become unfit for human consumption [6]. A severe attack by these pests will reduce the economic value of fruits and vegetables. It can cause yield losses from 30 to 100 %, depending on the host and season [7,8].

High reproductive rates, widespread distribution, and high egg production contribute to the extremely damaging nature of *Z. cucurbitae* [9]. *Zeugodacus cucurbitae* and *Bactrocera dorsalis* are two fruit fly species causing losses of US$ 200 million annually in Pakistan [5]. It can cause damage as much as 31.3 % and 28.6 % on the bitter gourd and watermelon respectively and yield loss of about 50 % in cucumber [10,11]. The melon fly infestation of bottle gourd was recorded at about 21 % [12]. Crop yields can be destroyed to the extent of 90 % by larvae that feed on the fruit [13]. The cucurbits are a major group of vegetables in Bangladesh which occupy 66.0 % of the land under vegetable production and contribute 11.0 % of total vegetable production.

Melon fly is also considered a devastating insect pest of cucurbits in Bangladesh [14]. Convincing efforts have previously been made to control *Z. cucurbitae* following integrated management techniques, including the use of insecticides and traps baited with lures and protein for targeting male flies, host plant resistance, field sanitation, and fruit bagging [15]. Globally most adopted control measures are biocontrol, cultural [16], botanicals [17], entomo-pathogenic fungi, viruses, bacteria, nematodes and chemical against melon fly. Despite this, using insecticides for *Z. curcubitae* is relatively inefficient because of the resistance development and cryptic feeding behaviour of larvae [8]. Chemical control of the melon fruit fly also has a risk for environmental negative effects. However, insecticides such as abamectin, cypermethrin, malathion, dichlorvos, phosphamidon, and endosulfan are moderately effective against the melon fly but not up to the mark that could suppress the notorious insect below the economic threshold value. Besides, the resistance of Spinosad was identified in the melon fly population in Hawaii [18]. Additionally, the chemical control of melon flies is very costly compared with the other management tactics.

Melon fly control with synthetic insecticides also comes with high risk to human health and the environment which warrants alternative strategies. Another option is to use conventional insecticides as they are always efficient but detrimental to non-target organisms, pollute water and soil, and lead to the development of pest resistance to pesticides that complicates pest management. Therefore, strategies that rely on the use of plant extracts or compounds with specific insect killing properties can enhance melon fly management whilst minimizing the negative impact on the environment and other non-target organisms. In addition, plant-derived insecticides could be an important part of sustainable agricultural pest management practices that protect crops from excessive losses and minimize agricultural ecological damage. It is consistent with increasing demands for environmentally sustainable pest control in the food systems. Considering this the botanicals, the application of eucalyptus leaf extract, parthenium leaf extract and neem seed extract was considered an effective botanical insecticide against *Z. cucurbitae* [19]*.* Also, neem oil, Cedar oil and pongamia oil gave potential results for managing this insect [20]. Despite this fact, the botanicals are rapid-acting but highly labile in the field [21].

Plants produce a variety of secondary metabolites which play an important role in affecting plant-insect interactions [22]. The use of plant metabolites as bio-insecticides could be an option and these could be advantageous in many ways i.e., does not cause any adverse effects to the environment, and cost-effective to the farmers and most importantly it is aligned with global sustainable development and food security initiative. Using plant essential oils, along with their bioactive constituents such as monoterpenes and sesquiterpenes, demonstrates notable efficacy as biorational insecticides against melon flies [23]. Diksha et al. found that the insecticidal properties of nerolidol (sesquiterpenes) can be attributed to its ability to modulate growth, immunomodulate, and cause cyto-genotoxicity, making it an attractive biopesticide to control *Z. cucurbitae* [23]. Chrysin was evaluated as one of the outstanding secondary plant metabolites which adversely affects the oviposition, growth and development of *Z. cucurbitae* [24]. Phloroglucinol inhibited the oviposition and the activity of detoxifying enzymes and antioxidants in this pest [25].

Competitional approach has advantages to screen potential metabolites which can be used as drug. Docking and other *in silico* approach aid the virtual screen to target protein and find solution with small molecules [25,26]. To address insecticide resistance and agricultural losses caused by melon fly infestations, we conducted a computational bioinformatics study to virtually screen phytocompounds with potential as sustainable insecticides. Our approach involved various bioinformatics tools and platforms, including molecular modeling, virtual docking, molecular dynamics simulation, *in silico* toxicity testing, and insecticidal likeliness analysis. While these methods provided valuable insights into potential phytocompounds for melon fly control, the study is limited by the absence of *in vivo* and field trials, which are necessary to fully evaluate efficacy and practical applicability.

## 2 Materials and methods

### 2.1 Retrieval of melon fly proteins and plant metabolites

The UniProtKB protein database (www.uniprot.org/) was searched for the retrieval of the melon fly hedgehog protein, spastin protein and ABC-type heme transporter ABCB6 protein. Furthermore, experimentally validated in various literature, a total of 59 plant metabolites with some insecticidal properties against different insects were identified (Supplementary Table 1) and their structures were retrieved from the PubChem server in SDF (3D) format, making a small library for evaluation against melon fly core proteins by molecular docking approach [27]. Then, OpenBabel v2.3 software was used to convert the SDF (3D) structures into PDB format for docking analysis preparation [28].

### 2.2 Secondary structure and conserved domain prediction

Secondary structures, or the shape that a protein's polypeptide backbone adopts, are made up of areas that are held together by hydrogen bonds between distinct atoms. CFSSP server (Chou and Fasman secondary structure prediction) was used to examine several secondary structural elements (such as helices, pleated sheets, and twists) [29] and PSIPRED (PSI-blast based secondary structure prediction) tools [30]. NCBI-CDD (NCBI Conserved Domains Database), a protein annotation resource of NCBI, was used to predict the conserved domain [31].

### 2.3 Tertiary structure prediction, refinement and validation

I-TASSER (Iterative Threading Assembly Refinement) was used to model all three proteins (hedgehog protein, spastin protein, and ABC-type heme transporter ABCB6) utilizing a hierarchical protein structure prediction technique [32]. I-TASSER employs a multi-stage process for protein structure prediction, starting with template search using LOMETS. Replica-exchange Monte Carlo simulations rebuild full-length models, while SPICKER identifies low-energy states. If no template is found, I-TASSER constructs the structure from scratch. The final model integrates spatial restraints from LOMETS and PDB structures via TM-align, representing the predicted protein structure. I-TASSER simulations yield decoys clustered by SPICKER. The top five models, reflecting the largest clusters, signify higher confidence. C-score quantifies model confidence, with lower-ranked models very uncommon surpassing higher-ranked ones in quality.

Protein structure prediction relies on target-template similarity, impacting model quality. GalaxyRefine, a GalaxyWEB server service, was used to eliminate undesirable mistakes in the predicted models [33]. The GalaxyRefine server utilizes a CASP10-tested method, rebuilding side chains and performing molecular dynamics simulation for structure enhancement. Demonstrating superior performance, it refines models from leading prediction servers, significantly improving both global and local structure quality.

The best-refined models of the studied proteins were determined through quality assessment and validation. Commonly used tools, ERRAT [34] and Molprobity [35] were employed to meet this purpose.

### 2.4 Screening of plant metabolites against melon fly proteins and analysis of insecticide surface hotspots

Molecular Docking is a useful tool for simulated screening of different compounds and determining how ligands inhibit their targets [36,37]. The PatchDock server (http://bioinfo3d.cs.tau.ac.il/PatchDock/) was used to determine the binding affinities of 59 plant metabolites with three melon fly proteins. The docking was performed with the help of shape based complementary principal of docking algorithm that provided by the patchdock server. The clustering RMSD remained as default 4.0 and protein–small ligand type was selected for complex [38]. Malathion (PubChem CID: 4004) was utilized as a positive control in this investigation as it has been shown to be chemically used to control melon fly [18,39]. The FireDock server (https://bioinfo3d.cs.tau.ac.il/FireDock/) was used to refine docked complexes; after refinement [40], the ligand binding complexes were subjected to further analysis using Discovery Studio v3.1 and the Pymol v2.0 server [40,41].

### 2.5 Molecular dynamics simulation analysis

The complexes of these ligands with the targets were subjected to molecular dynamics simulations to get insight into the binding free energies in a simulated biologically relevant environment. The Gromacs-2023 was used to run the 100 ns MD simulations [[42], [43], [44]]. The CHARMM-36 force field constants were used to generate protein topologies, while ligand topologies were obtained via the CGenFF website [45,46]. The complexes were solvated in a dodecahedron unit cell using the TIP3P water model [47] with a spacing of 1 nm between the unit cells and the complexes' borders. The resultant solvated systems were neutralized by sodium and chloride ions, which kept the molar concentration at 0.15-mol constant. The steepest descent energy reduction, with the threshold of the force constant set to 100 kJ mol-1 nm-1, alleviated the system's stearic conflicts. Following that, each system was subjected to two 1-ns equilibrations at constant temperature (NVT) and constant pressure (NPT). During NPT equilibration the constant temperature of 300 K was achieved with a modified Berendsen thermostat [48]; while during NPT equilibration the constant pressure of 1 atm was achieved with a Berendsen barostat [49]. However, during the 100 ns production phase MD simulations, the pressure conditions of 1 atm were achieved with the Parrinello-Rahman barostat [50], while the temperature of 300 K was achieved with a modified Berendensen thermostat. The position restraints on covalent bonds were achieved with the LINCS algorithm [51]. The long-range electrostatic energies were computed with the Particle Mesh Ewald (PME) method [52] with a cut-off of 1.2 nm. During NPT equilibration, a modified Berendsen thermostat [48] was used to maintain a constant temperature of 300 K, and a Berendsen barostat was used to maintain a constant pressure of 1 atm. During the 100 ns production phase MD simulations, however, pressure conditions of 1 atm were achieved using the Parrinello-Rahman barostat [50] and temperature conditions of 300 K were achieved using a modified Berendensen thermostat. The LINCS method was used to achieve location restrictions on covalent bonds [51]. The long-range electrostatic energies were calculated using the Particle Mesh Ewald (PME) method with a cut-off of 1.2 nm. The periodic boundary conditions were removed from the final MD simulation trajectories. The trajectories were examined for root mean square deviations (RMSD) in protein backbone atoms, root mean square fluctuation (RMSF) in side chain atoms, radius of gyration (Rg), and other parameters.

### 2.6 Insecticide profile and toxicity analysis of top metabolites

Insecticides often involve selective control of multiple pest species in a wide variety of crops following application to soil and/or plant foliage [53]. To achieve a balance of mobility and stability, an insecticide must diffuse through plant cell walls and transit through pest cuticles. It should also be able to withstand the effects of the environment, such as volatilization, wash-off, photolysis, and pyrolysis [54]. As a result, when compared to pharmaceutical medications, insecticides are likely to have drastically different bioavailability-related property profiles. The creation of new crop protectants can be accelerated by insecticide-likeness assessment, especially with the use of web resources, which can make the procedure more convenient. Using the InsectiPAD server, the pesticide-likeliness profile of top metabolites was evaluated [55]. Moreover, pkCSM, an online tool was prioritised to evaluate the relative toxicity of top metabolites [56]. The ProToxII server was utilized to predict the carcinogenicity and mutagenicity [57].

## 3 Results

### 3.1 Retrieval of melon fly proteins and plant metabolites

UniProt entry A0A0A1WD26 (532 amino acids), A0A0A1WSU7 (722 amino acids) and A0A0A1WT89 (850 amino acids) corresponding to hedgehog protein, spastin protein and ABC-type heme transporter ABCB6 protein respectively, were retrieved. PubChem ID, molecular weight, formula and source of retrieved 59 metabolites that contain insecticidal properties were listed in Supplementary Table 1.

### 3.2 Secondary structure and domain prediction

**Fig. 1:**
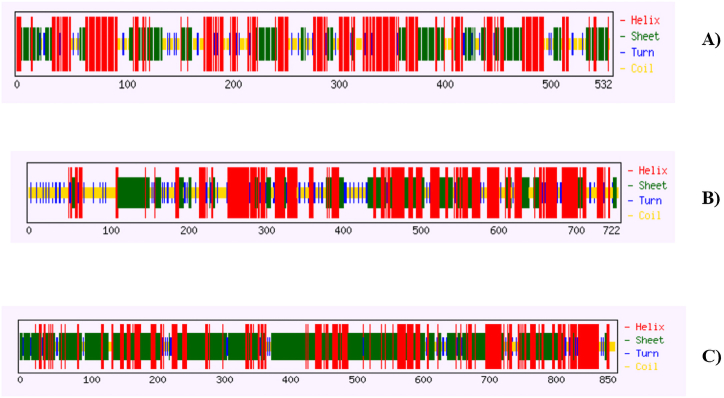
Predicted secondary structure of Hedgehog protein (A), Spastin protein (B), ABC-type heme transporter ABCB6 protein (C).

**Fig. 2:**
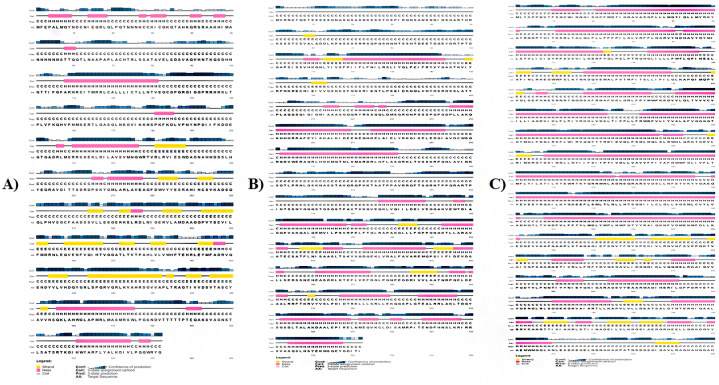
Predicted secondary structure's sequence-based chart of Hedgehog protein (A), Spastin protein (B), and ABC-type heme transporter ABCB6 protein (C).

**Fig. 3:**
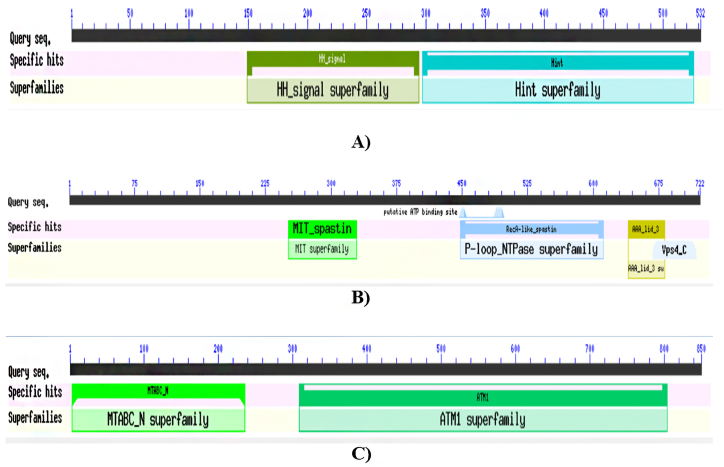
Predicted domains of Hedgehog protein (A), Spastin protein (B), and ABC-type heme transporter ABCB6 protein (C).

Results showed that hedgehog protein had 69.4 % (369 residues) helix, 39.3 % (209 residues) sheet and 11.3 % (60 residues) turn (Fig. 1A), while spastin protein showed to have 57.3 % (414 residues) helix, 31.0 % (214 residues) sheet and 15.9 % (115 residues) turn regions (Fig. 1B). Similarly, ABC-type heme transporter ABCB6 protein exhibited 77.8 % (661 residues) helix, 75.5 % (642 residues) sheet and 9.8 % (83 residues) turns (Fig. 1C). The predicted Secondary structure's sequence-based chart of hedgehog protein, spastin protein, and ABC-type heme transporter ABCB6 protein is shown in Fig. 2A–C. Higher numbers of α-helices suggest these proteins as thermostable (Fig. 2A–C). Protein annotation resource NCBI-CDD revealed that these hedgehog proteins have confined HH signal domain with a hint domain (Fig. 3A), while spastin protein showed to have MIT-spastin, Rec_A like spastin and AA lid 3 domain (Fig. 3B). Similarly, ABC-type heme transporter ABCB6 protein has MTABC-N and ATM1 superfamily domain (Fig. 3C–Table 1).

**Table 1:** Functional domains analysis of Melon fly proteins.

*Protein Name*	*Accession*	*Description*	*Interval*	*E-value*
Hedgehog protein	pfam01085	Hedgehog amino-terminal signalling domain	149–294	5.86e-108
	pfam01079	This is an alignment of the Hint module in the Hedgehog proteins	297–526	2.92e-85
Spastin protein	cd19524	The ATPase domain of spastin	448–612	2.20e-105
	cd02679	MIT: domain contained within Microtubule Interacting and Trafficking molecules.	251–329	1.65e-29
	pfam17862	AAA + lid domain	640–682	9.56e-09
	cl07827	Vps4 C terminal oligomerization domain	668–718	3.62e-05
ABC-type heme transporter ABCB6	COG5265	ABC-type transport system involved in Fe-S cluster assembly	309–804	0e+00
	pfam16185	Mitochondrial ABC-transporter N-terminal five TM region	3–236	1.71e-93

### 3.3 Tertiary structure prediction, refinement and validation

**Fig. 4:**
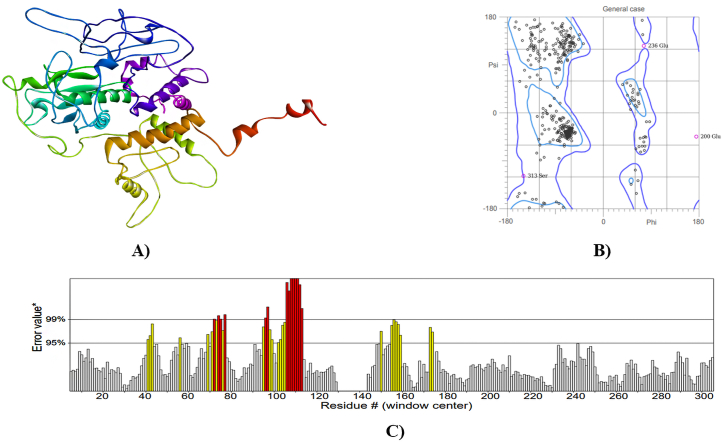
Homology model (A), Ramachandra plot assessment (B), ERRAT quality factor (C), of Hedgehog protein.

**Fig. 5:**
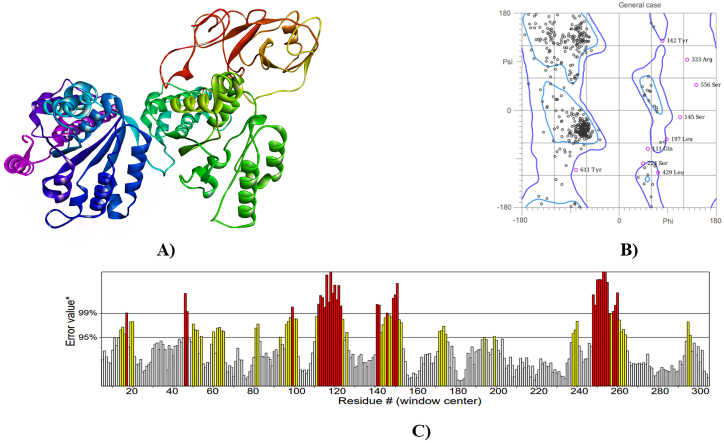
Homology model (A), Ramachandra plot assessment (B), ERRAT quality factor (C), of Spastin protein.

**Fig. 6:**
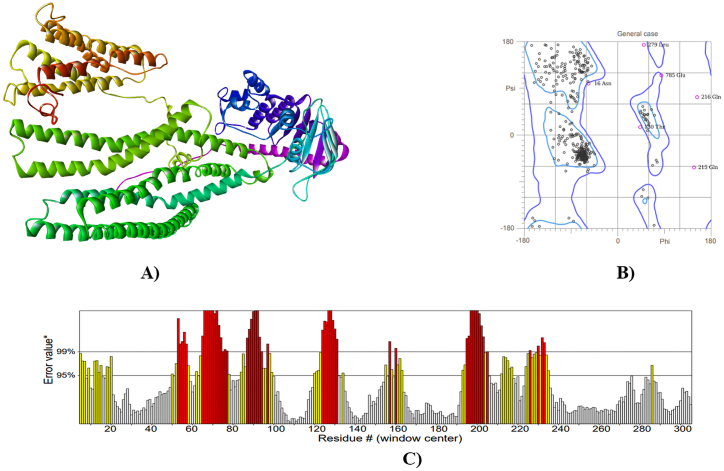
Homology model (A), Ramachandra plot assessment (B), ERRAT quality factor (C), of ABC-type heme transporter ABCB6 protein.

I-TASSER server performed three-dimensional homology modelling and predicted five models for each protein. The best models were identified based on C-Score (Fig. 4, Fig. 5, Fig. 6A), which showed overall quality factors 83.135 (hedgehog protein), 83.595 (spastin protein) and 86.010 (ABC-type heme transporter ABCB6 protein) at 0.01 and 0.05 level of significance (Fig. 4, Fig. 5, Fig. 6C). The C-score is the confidence score in I-TASSER evaluates model quality, derived from threading template alignments and structure assembly convergence parameters. A higher C-score indicates greater confidence in the predicted model's quality, and vice versa. The results of Ramachandran plot analysis revealed 86.4 % residues in favored, 98.3 % residues in allowed for hedgehog protein. The refined models of spastin protein and ABC-type heme transporter ABCB6 protein showed 88.3 % and 93.2 % residues in the favored region, while 97.3 % and 98.3 % residues were in the allowed region, respectively (Fig. 4, Fig. 5, Fig. 6B).

### 3.4 Screening of plant metabolites against melon fly proteins and analysis of insecticide surface hotspots

**Fig. 7:**
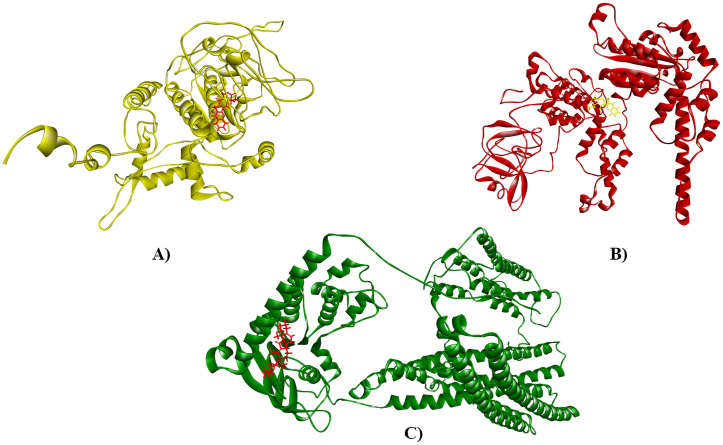
Molecular interaction of camptothecin with Hedgehog protein (A) and Spastin protein (B) and jervine with ABC-type heme transporter ABCB6 protein (C).

**Fig. 8:**
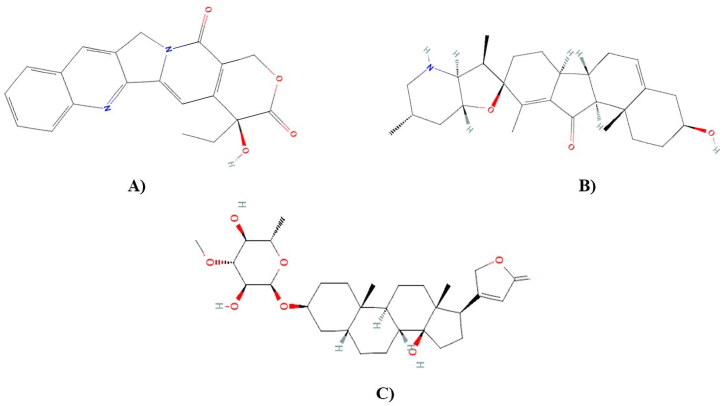
Chemical structure of top insecticide candidates, Camptothecin (A), Jervine (B), and Neriifolin (C).

**Fig. 9:**
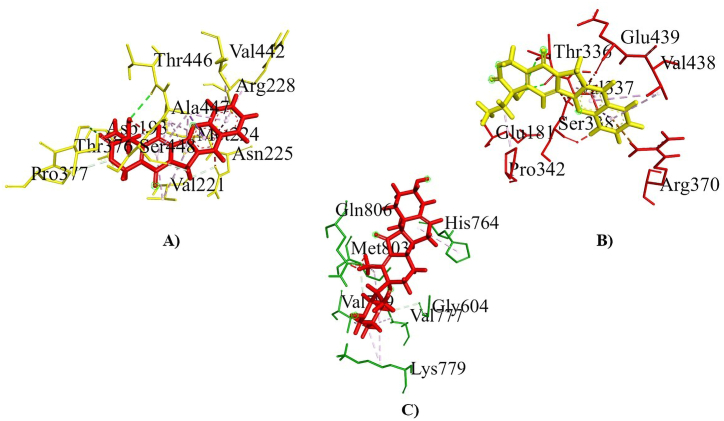
Ligand binding interaction of camptothecin with Hedgehog protein (A) and Spastin protein (B) and jervine with ABC-type heme transporter ABCB6 protein (C).

Molecular docking was used to examine the affinity between ligands and macromolecules using melon fly proteins (macromolecules) and plant metabolites (ligands) (Supplementary Table 2). The global binding energy of the top metabolites was used to rank them (Table 2). Notably, camptothecin, jervine and neriifolin showed a higher binding affinity with each of the three proteins and featured in the top list. Camptothecin showed maximum binding interaction with hedgehog protein (−57.32 kcal/mol; Fig. 7A) and spastin protein (−50.84 kcal/mol; Fig. 7B). While jervine showed the highest binding affinity with the ABC-type heme transporter ABCB6 protein (−43.92 kcal/mol; Fig. 7C). Neriifolin also experienced minimum binding energy with hedgehog protein (−52.71 kcal/mol), spastin protein (−37.16 kcal/mol) and ABC-type heme transporter ABCB6 protein (−43.89 kcal/mol) protein. The control malathion showed −30.25 kcal/mol, −26.25 kcal/mol and −28.17 kcal/mol for melon fly hedgehog protein, spastin protein and ABC-type heme transporter ABCB6 protein, respectively. The ligand binding interactions and structural conformations of each protein were investigated to unravel the insecticide surface hotspots. The chemical structure of top 3 metabolites is shown in Fig. 8A–C. The ligand binding interaction of camptothecin with Hedgehog protein, spastin protein and jervine with ABC-type heme transporter ABCB6 protein has been shown in Fig. 9A–C. Results revealed that the regions from 149 to 294 and 442–446 were crucial binding sites for hedgehog protein, where Arg144, His110, Lys148 and Val442 positions were most dominant (Table 2). Again, residues of 330–370 and 438–441 regions were identified as top surface hotspots for spastin protein. Moreover, two residues i.e. Pro342 and Arg370 were involved in forming the docked complexes in maximum cases. The ligands showed maximum binding affinity for the region of 600–814 amino acid sequences in the case of ABC-type heme transporter ABCB6 protein (Table 2).

**Table 2:** Analysis of global binding energy and interaction sites of the screened top 4 metabolites.

*Macromolecules*	*Ligands*	*Global Energy (Kcal/mol)*	*ACE*	*Score*	*Area*	*Ligand binding residues*
*Hedgehog Protein*	Camptothecin	−57.32	−16.68	4980	653.20	Val442, Thr446, Ala447, Ser448, Asn225, Met224, Val221, Asp193, Pro377, Arg228, Thr376
	Jervine	−56.74	−16.79	5792	751.90	Thr486, Trp475, Lys508, His511, Leu468, Cys449, Val441, Val442
	Neriifolin	−52.71	−15.99	6810	871.40	Arg145, His146, Arg147, Lys148, His110, Ala108, Leu76, Cys72, Ala68, Leu63
	Malathion (Control)	−30.25	−7.70	4752	599.60	Cys72, Arg144, Lys148, His110, Ser77
*Spastin Protein*	Camptothecin	−50.84	−13.70	4242	541.00	Pro342, Glu181, Ser338, Val337, Val438, Thr336, Glu439, Arg370
	Jervine	−40.31	−9.89	4844	564.30	Ser54, Arg233, Ala21, Tyr190, His191, Ala198
	Neriifolin	−37.16	−14.32	5402	747.20	Arg370, Lys443, Val438, Gly441, Glu439, Pro342, Glu181, Ile182, Asp183, Asn185
	Malathion (Control)	−26.25	−6.60	3946	446.40	Arg341, Pro342, Glu181, Thr336, Lys184, Asn185
*ABCB6 Protein*	Camptothecin	−36.00	−9.44	4418	545.20	Thr178, Val171, Ala174, Arg88, Ile100, Asn167
	Jervine	−43.92	−12.52	5280	616.50	Gln806, Met803, Val799, Val777, Lys779, Gly604, His764
	Neriifolin	−43.89	−9.03	6712	756.20	Asp814, Leu811, Thr735, His764, Met803, Gly604, Lys779
	Malathion (Control)	−28.17	−6.74	4058	519.30	Asp814, Leu811, Leu766, Ang765, Ala737, Thr735

### 3.5 Molecular dynamics simulation analysis

**Fig. 10:**
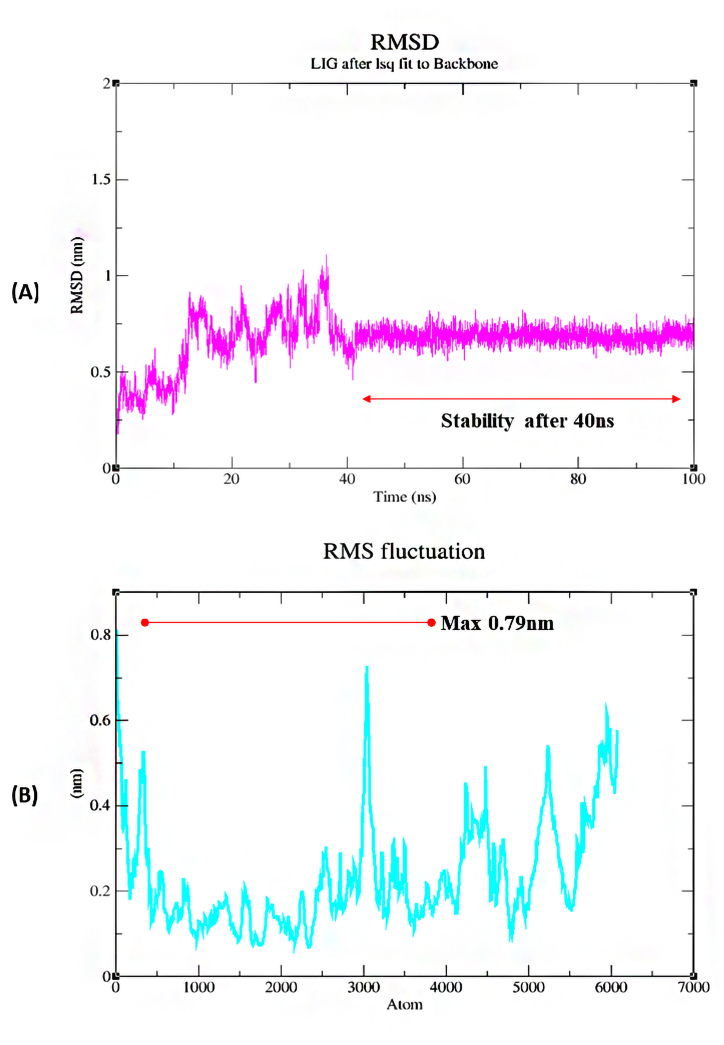
MD simulation analysis of Hedgehog protein with Camptothecin A) RMSD and B) RMSF.

**Fig. 11:**
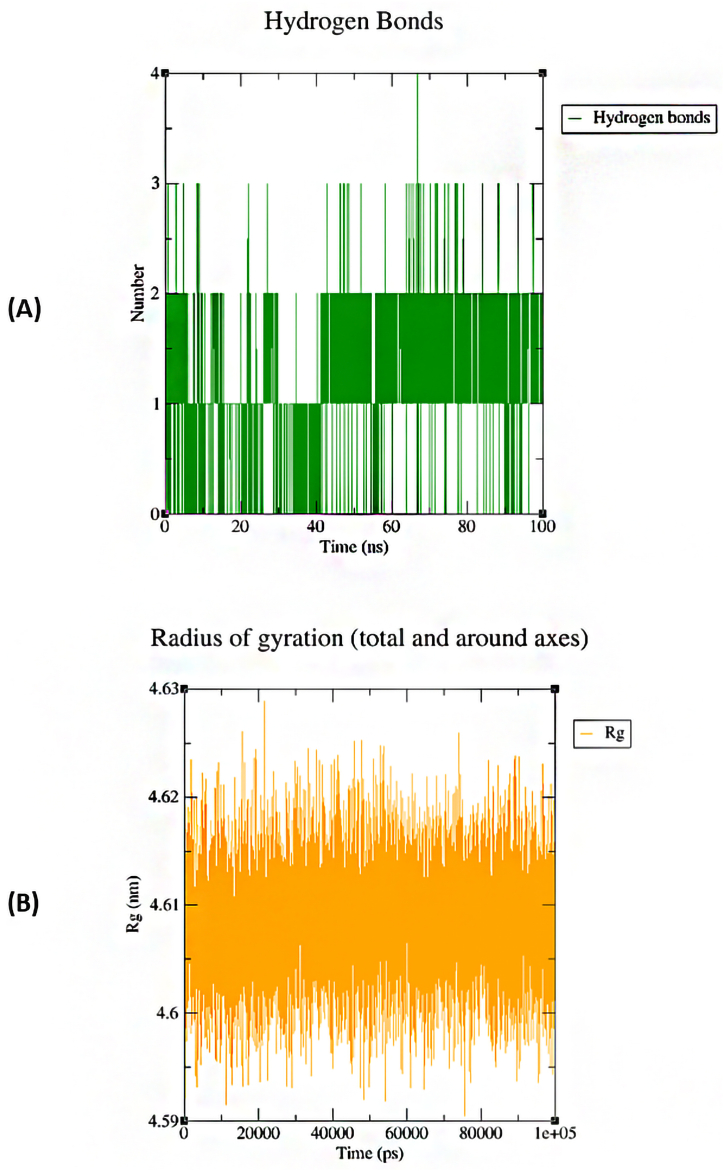
A) H-bond analysis of Hedgehog protein with Camptothecin and B) Rg of the complex.

The protein hedgehog with ligand camptothecin showed a significant complex in molecular docking analysis which is why the complex was selected for further MD simulation. The 100ns MD simulation was performed using GROMACS 2023 software. The RMSD of ligand fit proteins backbone showed a little fluctuation till 40ns. The highest RMSD was 1.2 nm and the lowest RMSD was 0.18 nm. The average RMSD was 0.7 nm. As the RMSD value of the complex was less than 1 nm. The complex could be preferred as completely stable at 100ns MD simulation analysis (Fig. 10A). The RMSF analysis of the complex also showed a significant result where the highest RMSF was 0.79 nm at atom index 3000. The overall RMSF of the complex was also less than 1 nm (Fig. 10B). Fig. 11 explains the H-bond interaction and Radius of gyration of the complex. The complex showed a maximum of 4 hydrogen bonds (Fig. 11A). The Rg (Radius of Gyration) showed the average result between 4.6 and 4.62 nm (Fig. 11B).

### 3.6 Insecticide profile analysis of top metabolites

**Fig. 12:**
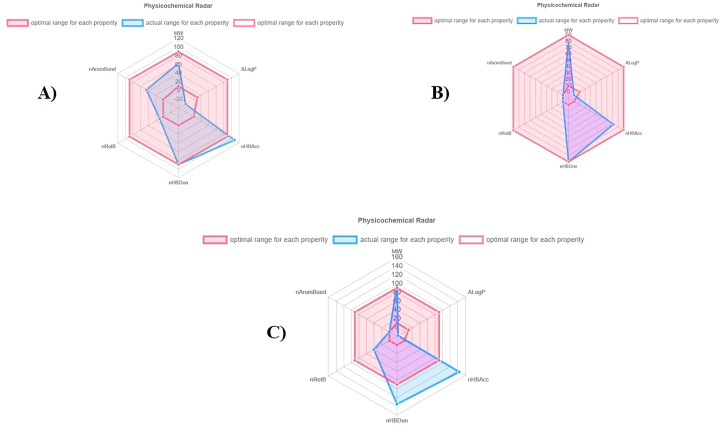
Physiochemical parameter radar of top insecticide candidates, Camptothecin (A), Jervine (B), and Neriifolin (C).

**Fig. 13:**
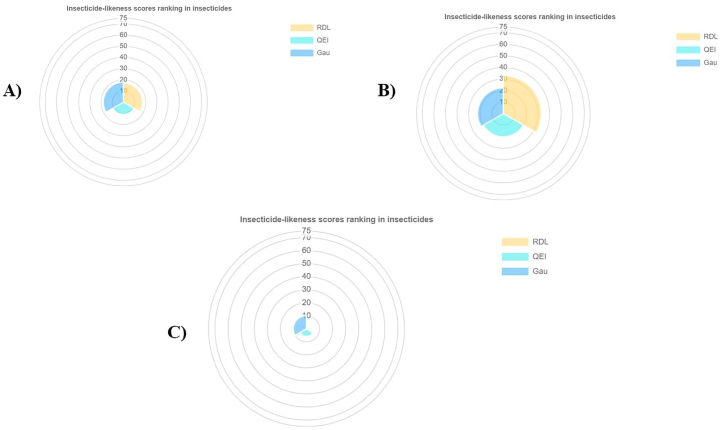
Insecticide likeliness score ranking in insecticides of top insecticide candidates, Camptothecin (A), Jervine (B), and Neriifolin (C).

**Fig. 14:**
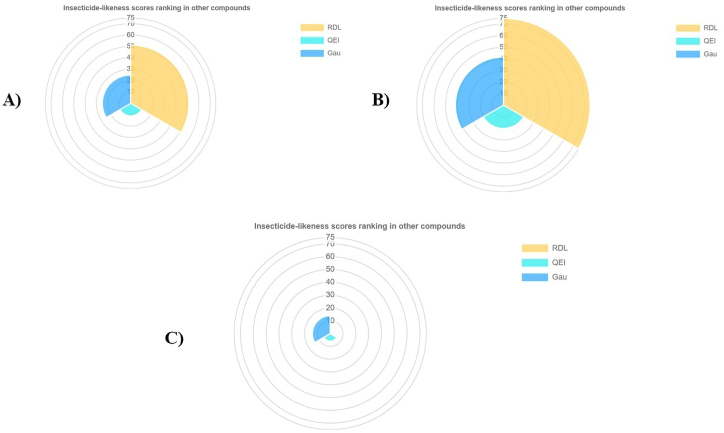
Insecticide likeliness score ranking in other compounds of top insecticide candidates, Camptothecin (A), Jervine (B), and Neriifolin (C).

Several insecticidal likeliness properties including physicochemical parameters, lipophilicity, geometric, water solubility, electrostatic and flexibility, photostability and topological and insecticide-likeness scores of top metabolites i.e., camptothecin, jervine, and neriifolin were demonstrated to evaluate their potential for insecticide fitness (Table 3). The physiochemical parameter radar of camptothecin, jervine, and neriifolin is shown in Fig. 12A–C. In this study, each insecticide candidate showed high lipophilicity with suitable geometry. The metabolites showed good electrostatic, flexibility and photostability phenomena. Neriifolin was found much more topologically polar than the other metabolites. The insecticide-likeness scores were found higher for jervine in both categories, such as among insecticides (Fig. 13B) and other compounds (Fig. 14B) in every algorithm. Camptothecin also produces a good insecticidal likeliness in both categories (Fig. 13, Fig. 14A) but the neriifolin found scoring significantly lower in the insecticide likeliness scoring algorithms for both categories (Fig. 13, Fig. 14C), respectively.

**Table 3:** InsectiPAD Insecticide profiling of top metabolites.

*Parameters*	*Camptothecin*	*Jervine*	*Neriifolin*
*Physicochemical Parameters*			
Molecular weight	349.119	425.293	534.319
Molar refractivity	92.665	118.571	135.2
Num. double bonds	10	3	2
Num. heavy atoms	26	31	38
Complexity of system	250.06	366.04	443.08
*Lipophilicity*			
ALogP	−1.025	−0.012	−0.871
MLogP	3	3.99	3.88
XLogP	0.02	1.88	2.941
*Geometric*			
PPSA-1	403.795	590.612	661.857
RPCS	1.06	3.913	0.645
THSA	462.446	644.807	620.651
RHSA	0.709	0.849	0.737
PNSA-1	248.51	169.221	180.824
RNCS	4.027	6.631	2.463
TPSA	189.858	115.026	222.03
RPSA	0.291	0.151	0.263
*Water Solubility*			
CLogS	−4.934	−4.663	−2.96
*Electrostatic and Flexibility*			
RPCG	0.134	0.105	0.083
RNCG	0.168	0.196	0.113
Num. Rot. bonds:	1	0	4
Frac. of Rot. bonds	0.033	0	0.093
*Photostability and Topological*			
Num. arom. bonds	7	0	0
Num. arom. atoms	6	0	0
Num. arom. rings	2	0	0
Num. rings	5	6	6
topoRadius	6	7	9
topoDiameter	11	14	18
TopoPSA	63.6	58.56	114.68
*Insecticide-likeness scores*			
RDL	0.895	1.418	0
QEI	0.214	0.393	0.115
Gau	3.176	3.932	1.794

### 3.7 Toxicity pattern analysis of top metabolites

The relative toxicity (i.e., Carcinogenicity, Mutagenicity, AMES toxicity, oral rat toxicity, skin sensitization and *T. pyriformis* toxicity) of top metabolites were predicted via pkCSM server and ProTox II server. The results revealed negative outcomes in the AMES test, Carcinogenicity, Mutagenicity, AMES toxicity and skin sensitization inhibitor for all the candidates. The top insecticide candidates did not interact with hERGI (human ether-a-go-go-related gene I), confirming that they wouldn't contribute to any human heart-related complexities. Minnow Toxicity values of all metabolites were greater than −0.3 log mM indicating them as non-toxic. Besides, oral rat acute toxicity (LD50) and *T. pyriformis* toxicity did not show any undesirable effects by the top insecticide candidates that could reduce their insecticidal-likeness properties (Table 4).

**Table 4:** Toxicity properties analysis of screened insecticide candidates.

*Toxicity properties*	*Camptothecin*	*Jervine*	*Neriifolin*
Carcinogenicity	No	No	No
Mutagenicity	No	No	No
AMES toxicity	No	No	No
hERG I inhibitor	No	No	No
Oral Rat Acute Toxicity (LD_50_) mol/Kg	2.565	2.513	2.508
Skin Sensitization	No	No	No
T.Pyriformis toxicity (log ug/L)	0.298	0.289	0.287

## 4 Discussion

In the absence of efficient biological or chemical management, the melon fly is becoming a threat to food security. Melon fly larvae can be found in flowering plants, fruits, and various plant sections. Ovipositional harm can occur even if the larvae are unable to survive because it allows germs to penetrate the fruit or causes deformities in the growing fruit. Chemical insecticides such as dichlorvos, phosphamidon, and endosulfan have been used to manage melon fly destruction in the past. However, these pesticides do not meet the required standards. They also have a wide spectrum of harmful side effects [39]. Malathion has a moderate level of efficacy against melon flies, according to many studies [58]. Malathion (PubChem CID: 40004) was chosen as a positive control in our investigation for the targeted proteins because of its effectivity against melon flies.

Hedgehog protein, spastin, and ABC-type heme transporter ABCB6 protein were the three key proteins addressed in melon flies. Hedgehog is involved in the development of larval body segments as well as the production of adult appendages. Typically, paired appendages around the mouth are used to collect and handle food, and they are usually tailored according to the animal's diet. Animal appendages are protrusions from the body wall that serve a variety of purposes, including mobility, grooming, and feeding [59]. Spastin is a microtubule-severing protein that may work by generating new microtubule polymerization seeds. Microtubules are significant cellular components of the insect cuticle's mechanosensory and chemosensory sensilla, and a variety of ideas have been presented to explain their role in sensory transduction. Chemicals that disrupt microtubules, like as colchicine and vinblastine, also interfere with transduction in these sensilla, which has been linked to their anti-microtubule action [60]. In tick midgut cells, the ATP Binding Cassette Transporter (ABC) mediates both heme and pesticide detoxification [61]. By inhibiting this protein, the insect may become more sensitive to pesticides. CpMRP (ABC subfamily C) of *C. populi* works as a pacemaker, moving particular metabolites from the hemolymph to defence secretions and finally excreting them from the body, according to a study. CpMRP silencing renders larvae vulnerable, showing that CpMRP is involved in secretion [62].

The use of computer-aided inhibitor design has helped to speed up the development of inhibitors for a variety of pest problems. These methods can be used to investigate the potential of potent small compounds as ligands/inhibitors [[63], [64], [65]]. Analyzing the interactions between macromolecules and tiny ligands is a good technique to streamline the contemporary inhibitor discovery process [65]. This reduces the amount of time and money spent on the inhibitor development process. Following that, we used molecular docking-based virtual screening to assess several plant-based bioactive compounds as effective melon fly protein inhibitors *in silico*. The top hit compounds in terms of minimum global binding energy were camptothecin, jervine, and neriifolin, according to the current results. Comparing with the control malathion showed only −30.25 kcal/mol, −26.25 kcal/mol and −28.17 kcal/mol for melon fly hedgehog protein, spastin protein and ABC-type heme transporter ABCB6 protein, respectively. Our top metabolites outperformed the chemical in case of all the targets and showed a significant result.

The ligand binding interactions and structural conformations of each protein were investigated to unravel the insecticide surface hotspots. The regions 149–294 and 442–446 were found to be critical binding sites for hedgehog protein, with Arg144, His110, Lys148, and Val442 being the most prominent. This region contains the HH signal domain, which is conserved throughout the protein. This domain is needed for embryonic cell differentiation, and blocking this region prevents embryonic cell differentiation in melon flies. The top surface hotspots for spastin protein were discovered at residues 330–370 and 438–441, where it incorporates the rec A domain, which is an ATPase binding domain involved in microtubule dynamics. Furthermore, in the majority of instances, two residues, Pro342 and Arg370, were involved in the docking complex formation. In the scenario of the ABC-type heme transporter ABCB6 protein, the ligands had the highest binding affinity for a region of 600–814 amino acid sequences (Fig. 9C). Similarly, the MTABC N domain contains an ABC-type transport system that is involved in Fe-S cluster assembly, as well as permease and ATPase components. The Hedgehog protein with camptothecin complex showed a significant binding affinity. Thus, this complex was further evaluated in 100ns MD simulation analysis. In the MD simulation run the complex showed a little fluctuation in the beginning but after 40ns the complex showed significant stability in the RMSD of ligand with backbone of the protein. The RMSF analysis also showed no major fluctuation in the MDs run. Overall the complex showed a good binding stability in the current study. Each pesticide candidate in this investigation had a high lipophilicity and a good geometry. Electrostatic, flexibility, and photostability were all good in the metabolites. The topological polarity of Neriifolin was substantially higher than those of the other metabolites. The ability of pesticide molecules to penetrate cell membranes and have a negative effect on cells and the organism as a whole. Higher lipophilicity facilitates the passage of a substance across lipid cell membranes and may also indicate its toxicity towards the insect [66]. All the proposed candidates showed up with MlogP less than 5 which means they have good absorption and permeability towards the target organism. The geometry of insecticides is highly impactful in an insecticide characteristic such as all candidates have a high number of PPSA-1 which indicates they are highly electropositive referring to their high possibility of reactivity [67]. Sometimes highly insecticidal action of an insecticide cannot last because of its lower photostability [68]. The photostability and topology of the insecticides contribute to their structural features which ultimately contribute to their fate in the environment [69]. The candidates showed low water solubility and low water-soluble systemic insecticides have a longer residual activity [70]. A lower number of rotatable bonds in a molecule would result in a very positive effect on its bioavailability [71]. Camptothecin and jervine will be more bioavailable for the target than neriifolin, in another way they will be more effective than neriifolin in melon fly control. In every algorithm, the insecticide-likeness scores for jervine were greater in both categories (among insecticides and other chemicals). In both areas, the camptothecin produced a good insecticidal likeliness, while the neriifolin scored significantly lower in the insecticide likeliness scoring algorithms. Moreover, the synthetic insecticide use by farmers raises the issue of the risks that these products pose to man and the environment as they come into contact, to clarify the status of our proposed natural insecticide candidate's safety these tests were done. Our natural insecticide candidates were safe in terms of creating genetic mutation proved by the negative outcome of AMES and mutagenicity test; chemicals could also act as cancer-inducing carcinogens where our natural insecticides pose negative outcome in carcinogenicity test means they were not carcinogenic agents also they did not create any kind of skin sensitization as they pose negative result in skin sensitization test. The leading insecticide options against melon fly showed no negative effects that could impair their insecticidal-likeness properties. "Pesticide-likeness" adapts the concept of "drug-likeness" from pharmaceuticals to pesticide research [32]. Computer-aided drug design (CADD) enhances drug discovery efficiency and cost-effectiveness [72]. Nevertheless, computational models encounter challenges, such as difficulties in accurately representing complex biological systems and the persistent need to improve virtual screening hit rates [72,73]. Furthermore, one important limitation to computational methods, however, is that their applicability is limited to static structures of similar scaffolds while neglecting the dynamical nature of the ligands [74]. This study does not account for environmental and ecological factors which is the key limitation for the computational approach, highlighting the importance of *in vitro* and field trials to accurately assess the efficacy and potential impacts of the identified phytocompounds. Such trials would provide a comprehensive evaluation under stressed and *in vivo* conditions. Certain plant secondary metabolites like alkaloids, polyphenols can be toxic to animals, prompting herbivores to consume less based on metabolite concentration [[75], [76], [77], [78]]. Appropriate dosage for plant application needs further *in vitro* and field testing. This computational study identifies potential metabolites for melon fly control, an initial step toward developing eco-friendly, natural alternatives to synthetic insecticides [79]. Hoesain et al. outlined methodologies for evaluating plant metabolites as insecticides, offering a framework that can be used in future field studies [79]. Nevertheless, this research offers a foundation for further exploration of the top metabolites and may encourage researchers to investigate these candidates for developing sustainable melon fly control methods.

## 5 Conclusion

The study of plant metabolites as possible insecticides against *Zeugodacus cucurbitae* demonstrates their efficiency in disrupting the melon fly core protein's function while avoiding environmental damage. Identification of controlling agent using computational methods can save time and trial cost for the experimental validation. According to the findings, jervine and camptothecin could be the effective melon fly control agents, preventing melon fly-associated economic loss. In addition, the AMES toxicity analysis showed none of them negative or toxic result, which indicates that the plant metabolites could be a potential alternative to the harsh chemical that conventionally used to control melon fly. However, future research is needed for *in-vitro* validation and to find out the optimal delivery systems of the proposed insecticides for field trial. In the future, procedures will be refined and interdisciplinary collaboration will be encouraged to promote practical incorporation into agroecosystems, hence increasing the sustainability of pest management tactics.

## CRediT authorship contribution statement

**Zinat Jahan Chowdhury:** Writing – original draft, Validation, Resources, Project administration, Data curation, Conceptualization, Investigation, Methodology, Software, Visualization. **Anik Banik:** Writing – original draft, Visualization, Software, Resources, Methodology, Investigation, Formal analysis, Data curation, Project administration, Validation. **Tanjin Barketullah Robin:** Writing – review & editing, Visualization, Software, Formal analysis, Validation. **Mohammed Rashed Chowdhury:** Writing – review & editing, Visualization, Supervision, Conceptualization, Project administration, Validation.

## Ethics approval

Not applicable.

## Data and code availability statement

Data included in the article/supplementary material is referenced in the article.

## Declaration of generative AI in scientific writing

The authors did not use any artificial intelligence assisted technologies in the writing process.

## Funding information

This research did not receive any specific grant from funding agencies in the public, commercial, or not-for-profit sectors.

## Declaration of competing interest

The authors declare that they have no known competing financial interests or personal relationships that could have appeared to influence the work reported in this paper.
