# Supplementary material for: Deciphering the potential of plant metabolites as insecticides against melon fly (Zeugodacus cucurbitae): Exposing control alternatives to assure food security
Source: Heliyon. 2025 Jan 16;11(2):e42034. doi: 10.1016/j.heliyon.2025.e42034 (PMC11791130; doi:10.1016/j.heliyon.2025.e42034)
Supplement: Multimedia component 1 [file mmc1.docx]

**Supplementary Table 1:** List of Plant metabolites with insecticidal activity.

| ***Metabolites*** | ***PubChem ID*** | ***Chemical type*** | ***Plant source*** | ***Molecular formula*** | ***Molecular weight*** | ***Remarks*** |
| --- | --- | --- | --- | --- | --- | --- |
| A pinene | 6654 | Terpenoids | *Teucrium montanum* | C10H16 | 136.23 | Shahriari et al., 2016 |
| A-terpineol | 17100 | Terpenoids | *Nepeta nepetella* | C10H18O | 154.25 | Tong and coats, 2012 |
| A-thujone | 12304613 | Terpenoids | *Tanacetum vulgare* | C10H16O | 152.23 | Hwang et al., 1985 |
| Anabasine | 2181 | Alkaloids | *nicotiana* | C10H14N2 | 162.23 | Roark.,1947 |
| Anethole | 637563 | Phenolic compounds | *Erucaria microcarpa* | C10H12O | 148.20 | Chang and Ahn., 2002 |
| Aconitine | 245005 | Alkaloids | *Aconitum napellus* | C34H47NO11 | 645.7 | Wei et al., 2019 |
| Absinthin | 442138 | Terpenoids | *Artemisia sieversiana* | C30H40O6 | 496.6 | Da at al., 2005 |
| Aphylline | 442940 | Alkaloids | *Leptolobium panamense* | C15H24N2O | 248.36 | Bermúdez at al., 2019 |
| Anonaine | 160597 | Alkaloids | *Xylopia emarginata* | C17H15NO2 | 265.31 | Mamun and Ahmed., 2011 |
| Ascaridole | 10545 | Terpenoids | *Athamanta macedonica* | C10H16O2 | 168.23 | Urzua et al., 2010 |
| Apiole | 10659 | Terpenoids | *Niphogeton dissecta* | C12H14O4 | 222.24 | Babri et al., 2012 |
| Absinthin | 442138 | Terpenoids | *Artemisia sieversiana* | C30H40O6 | 496.6 | Da et al., 2005 |
| B asarone | 5281758 | Terpenoids | *Asarum hypogynum* | C12H16O3 | 208.25 | Ascher et al., 1989 |
| Bergapten | 2355 | Phenolic compounds | *Ficus virens* | C12H8O4 | 216.19 | Yajima and Munakata., 1979 |
| Caffeine | 2519 | Alkaloids | *Theobroma cacao* | C8H10N4O2 | 194.19 | Araque et al., 2007 |
| Camptothecin | 24360 | Alkaloids | *Camptotheca acuminata* | C20H16N2O4 | 348.4 | Liu et al., 2010 |
| Caryophyllene-oxide | 1742210 | Terpenoids | *Xylopia emarginata* | C15H24O | 220.35 | Cárdenas et al., 2015 |
| Cinerins | 5371326 | Terpenoids | *Tanacetum cinerariifolium* | C20H28O3 | 316.4 | Sawicki et al., 1962 |
| Carvone | 7439 | Terpenoids | *Teucrium polium* | C10H14O | 150.22 | Franzios et al., 1997 |
| Cinnamaldehyde | 637511 | Terpenoids | *Cinnamomum ceylanicum* | C9H8O | 132.16 | Lu et al., 2020 |
| Celabenzine | 442847 | Alkaloids | *Gymnosporia mossambicensis* | C23H29N3O2 | 379.5 | Rochais et al., 2010 |
| Deguelin | 107935 | Phenolic compounds | *Tephrosia vogelii* | C23H22O6 | 394.4 | Caboni et al.,2004 |
| Delcosine | 145925533 | Alkaloids | *Aconitum barbatum* | C24H39NO7 | 453.6 | Davidson.,1929 |
| Dillapiol | 10231 | Phenolic compounds | *Asarum yaeyamense* | C12H14O4 | 222.24 | Bernard et al., 1995 |
| Estragole | 8815 | Terpenoids | *Vitis rotundifolia* | C10H12O | 148.20 | Ling et al., 2009 |
| Delcoline | 441727 | Alkaloids | *Aconitum barbatum* | C25H41NO7 | 467.6 | Davidson.,1929 |
| Elemicin | 10248 | Phenolic compounds | *Anemopsis californica* | C12H16O3 | 208.25 | Tafurt and Muñoz., 2018. |
| Eugenol | 3314 | Terpenoids | *Cinnamomum verum* | C10H12O2 | 164.20 | Fernandes et al.,2020 |
| Galanthamine | 9651 | Alkaloids | *Galanthus nivalis* | C17H21NO3 | 287.35 | Maazoun et al., 2017 |
| Gramine | 6890 | Alkaloids | *Murraya exotica* | C11H14N2 | 174.24 | Lu et al., 2019 |
| Helenalin | 23205 | Terpenoids | *Psilostrophe cooperi* | C15H18O4 | 262.30 | Adams and herz., 1949 |
| Himachalol | 121536 | Terpenoids | *Tilia tomentosa* | C15H26O | 222.37 | Singh and agarwal., 1988. |
| Glaucarubinone | 441796 | Terpenoids | *Simarouba amara* | C25H34O10 | 494.5 | Polonsky et al., 1989 |
| Isopimpinellin | 68079 | Phenolic Compound | *Zanthoxylum mayu* | C13H10O5 | 246.21 | Miyazawa et al., 2004 |
| Limonene | 22311 | Terpenoids | *Teucrium montanum* | C10H16 | 136.23 | Hebeish et al., 2008 |
| Menthone | 26447 | Terpenoids | *Xylopia aromatica* | C10H18O | 154.25 | Franzios et al., 1997 |
| Jervine | 10098 | Alkaloids | *Veratrum stamineum* | C27H39NO3 | 425.6 | Stankovic et al., 2020 |
| Myristicin | 4276 | Terpenoids | *Chaerophyllum azoricum* | C11H12O3 | 192.21 | Reed et al., 1982 |
| Mesaconitine | 441747 | Alkaloids | *Aconitum napellus* | C33H45NO11 | 631.7 | Xiaodong et al., 2009 |
| Nicotine | 89594 | Alkaloids | *Nicotiana tabacum* | C10H14N2 | 162.23 | Matsuda et al., 2001 |
| Napelline | 441749 | Alkaloids | *Aconitum karakolicum* | C22H33NO3 | 359.5 | Chen et al., 2015 |
| Myosmine | 442649 | Alkaloids | *Nicotiana tabacum* | C9H10N2 | 146.19 | Yamamotoand Kamimura., 1963 |
| Methyl-chavicol | 70235324 | Phenolic compounds | *Hamamelis virginiana* | C19H28O | 272.4 | De menezes et al., 2020 |
| Neriifolin | 441867 | Terpenoids | *Cerbera manghas* | C30H46O8 | 534.7 | Reed et al., 1982 |
| Picrotoxinin | 442292 | Terpenoids | *Anamirta cocculus* | C15H16O6 | 292.28 | Miller et al., 1979 |
| Physostigmine | 5983 | Alkaloids | *Physostigma venenosum* | C15H21N3O2 | 275.35 | Murao and Hayashi., 1986. |
| Piperitenone-oxide | 61942 | Terpenoids | *Calamintha incana* | C10H14O2 | 166.22 | Kasrati at al 2015 |
| Plumbagin | 10205 | Phenolic compounds | *Drosera auriculata* | C11H8O3 | 188.18 | Sreelatha et al., 2010 |
| Psoralen | 6199 | Phenolic compounds | *Citrus bergamia* | C11H6O3 | 186.16 | Francisco et al.,2013 |
| Pulegone | 442495 | Terpenoids | *Nepeta cataria* | C10H16O | 152.23 | Kasrati et al., 2015 |
| Ricinine | 10666 | Alkaloids | *Ricinus communis* | C8H8N2O2 | 164.16 | De melo et al., 2009 |
| Podophyllotoxin | 11384429 | Phenolic compounds | *Podophyllum peltatum* | C22H22O8 | 414.4 | Wang et al., 2012 |
| Sparteine | 7014 | Alkaloids | *Lupinus mutabilis* | C15H26N2 | 234.38 | Fowsiya and Madhumitha., 2020. |
| Thymol | 6989 | Terpenoids | *Xylopia aromatica* | C10H14O | 150.22 | Kordali et al.,2008 |
| Sesamin | 72307 | Phenolic compounds | *Ageratum conyzoides* | C20H18O6 | 354.4 | Haller et al., 1942 |
| Rotenone | 6758 | Phenolic compounds | *derris trifoliata* | C23H22O6 | 394.4 | Gosálvez.,1983 |
| Piperine | 638024 | Alkaloids | *Piper nigrum* | C17H19NO3 | 285.34 | Paula et al., 2000 |
| Linalool | 6549 | Terpenoids | *Ocimum basilicum* | C10H18O | 154.25 | Beier et al., 2014 |
| Ephedrine | 9294 | Alkaloids | *Ephedra sinica* | C10H15NO | 165.23 | Wu et al., 1988 |

**Supplementary Table 2:** Molecular Docking between Targeted proteins and Plant metabolites.

| ***Macromolecules*** | ***Ligands/ Metabolites*** | ***Global energy***  ***(Kcal/mol)*** | ***ACE*** | ***Score*** | ***Area*** |
| --- | --- | --- | --- | --- | --- |
| ***Hedgehog Protein*** | A Pinene | -23.69 | -6.39 | 3074 | 356.80 |
|  | A Terpineol | -25.83 | -5.86 | 3272 | 404.90 |
|  | A Thujone | -26.06 | -5.92 | 3094 | 386.60 |
|  | Aconitine | -42.81 | -5.33 | 7850 | 971.90 |
|  | Anabasine | -29.57 | -7.97 | 3542 | 406.00 |
|  | Absinthin | -39.88 | -12.93 | 6116 | 792.80 |
|  | Anonaine | -37.62 | -6.11 | 4280 | 523.80 |
|  | Anethole | -28.19 | -6.06 | 3342 | 370.00 |
|  | Aphylline | -39.22 | -8.74 | 4022 | 503.60 |
|  | Ascaridol | -26.50 | -5.60 | 3358 | 412.00 |
|  | Asarinin. | -43.85 | -10.82 | 5190 | 547.80 |
|  | B Asarone | -26.27 | -6.83 | 3972 | 446.20 |
|  | Apiole | -30.93 | -7.44 | 3976 | 482.80 |
|  | Bergapten | -32.00 | -5.59 | 3558 | 395.10 |
|  | Carvone | -25.60 | -4.41 | 3326 | 373.40 |
|  | Camptothecin | -57.32 | -16.68 | 4980 | 653.20 |
|  | Cinerins | -39.74 | -11.02 | 5344 | 636.80 |
|  | Cinnamaldehyde | -25.31 | -5.97 | 2978 | 368.00 |
|  | Delcoline | -50.28 | -12.77 | 5612 | 755.90 |
|  | Delcosine | -48.14 | -10.39 | 5346 | 741.40 |
|  | Dillapiol | -30.07 | -5.81 | 3966 | 495.10 |
|  | Elemicin | -30.92 | -8.09 | 4228 | 515.80 |
|  | Ephedrine | -28.68 | -8.07 | 3484 | 455.30 |
|  | Estragole | -26.96 | -6.10 | 3358 | 417.40 |
|  | Galanthamine | -38.45 | -8.80 | 4216 | 539.60 |
|  | Helenalin | -31.97 | -6.20 | 3944 | 490.40 |
|  | Himachalol | -30.06 | -5.63 | 3948 | 496.40 |
|  | Glaucarubinone | -40.40 | -10.38 | 5968 | 780.80 |
|  | Isopimpinellin | -34.67 | -9.60 | 3908 | 420.40 |
|  | Limonene | -25.88 | -4.14 | 3206 | 374.00 |
|  | Caffeine | -27.92 | -6.94 | 3428 | 428.30 |
|  | Linalool | -26.55 | -7.37 | 3586 | 428.40 |
|  | Mesaconitine | -38.15 | -11.44 | 7098 | 992.10 |
|  | Methyl Chavicol | -42.79 | -9.67 | 4806 | 603.90 |
|  | Myristicin | -28.06 | -6.98 | 3856 | 442.40 |
|  | Myosmine | -30.47 | -10.48 | 3098 | 380.10 |
|  | Napelline | -42.37 | -10.07 | 4906 | 618.60 |
|  | Neriifolin | -52.71 | -15.99 | 6810 | 871.40 |
|  | Nicotine | -28.75 | -6.05 | 3342 | 384.80 |
|  | Physostigmine | -38.38 | -9.86 | 4730 | 589.60 |
|  | Picrotoxinin | -36.65 | -8.85 | 3890 | 473.10 |
|  | Plumbagin | -29.05 | -7.36 | 3238 | 385.90 |
|  | Podophyllotoxin | -45.44 | -11.03 | 5830 | 727.10 |
|  | Piperine | -38.37 | -8.99 | 4788 | 602.90 |
|  | Piperitenone Oxide | -28.03 | -5.36 | 3310 | 375.10 |
|  | Ricinine | -26.25 | -5.83 | 3146 | 383.40 |
|  | Rotenone | -48.60 | -11.41 | 5684 | 734.10 |
|  | Sparteine | -39.03 | -11.20 | 3870 | 447.80 |
|  | Sesamin | -45.74 | -9.12 | 5508 | 665.40 |
|  | Thymol | -27.72 | -5.84 | 3366 | 368.30 |
|  | Caryophyllene Oxide | -31.11 | -8.73 | 3764 | 424.80 |
|  | Jervine | -56.74 | -16.79 | 5792 | 751.90 |
|  | Gedunine | -27.09 | -7.00 | 3650 | 397.60 |
|  | Celabenzine | -48.60 | -10.21 | 6108 | 755.20 |
|  | Gramine | -30.12 | -7.44 | 3408 | 402.90 |
|  | Deguelin | -47.53 | -13.24 | 5460 | 707.50 |
|  | Menthone | -24.13 | -5.87 | 3418 | 440.40 |
|  | Psoralen | -30.07 | -7.59 | 3610 | 452.40 |
|  | Pulegone | -28.26 | -5.66 | 3382 | 370.50 |
|  | Malathion | -30.25 | -7.70 | 4752 | 599.60 |

| ***Macromolecules*** | ***Ligands/ Metabolites*** | ***Global energy***  ***(Kcal/mol)*** | ***ACE*** | ***Score*** | ***Area*** |
| --- | --- | --- | --- | --- | --- |
| ***Spastin Protein*** | A Pinene | -25.24 | -7.19 | 2656 | 279.50 |
|  | A Thujone | -26.14 | -6.17 | 2830 | 338.60 |
|  | Absinthin | -36.73 | -10.49 | 5224 | 667.80 |
|  | Aconitine | -42.64 | -6.37 | 6222 | 742.50 |
|  | Anabasine | -32.95 | -9.16 | 2970 | 315.90 |
|  | Anethole | -30.76 | -9.75 | 3056 | 382.70 |
|  | Aphylline | -35.23 | -11.38 | 3630 | 403.50 |
|  | Anonaine | -35.96 | -4.64 | 3830 | 493.80 |
|  | Apiole | -30.31 | -9.49 | 3684 | 468.30 |
|  | Ascaridol | -25.17 | -7.90 | 3004 | 324.70 |
|  | B Asarone | -35.82 | -7.60 | 3656 | 459.70 |
|  | Caffeine | -29.29 | -8.89 | 3070 | 340.60 |
|  | Bergapten | -36.37 | -9.70 | 3252 | 368.30 |
|  | Camptothecin | -50.84 | -13.70 | 4242 | 541.00 |
|  | Celabenzine | -45.36 | -15.08 | 4698 | 555.40 |
|  | Carvone | -28.21 | -7.15 | 2998 | 360.20 |
|  | Cinnamaldehyde | -25.58 | -7.33 | 2768 | 289.50 |
|  | Deguelin | -38.63 | -10.83 | 4692 | 629.90 |
|  | Cinerins | -31.68 | -7.59 | 4516 | 516.40 |
|  | Delcoline | -38.59 | -10.26 | 4966 | 592.00 |
|  | Delcosine | -33.75 | -9.61 | 4688 | 548.00 |
|  | Dillapiol | -27.17 | -9.26 | 3512 | 399.10 |
|  | Gedunine | -29.73 | -8.74 | 3214 | 392.70 |
|  | Ephedrine | -30.94 | -8.16 | 2980 | 322.50 |
|  | Galanthamine | -39.67 | -12.29 | 3926 | 454.60 |
|  | Elemicin | -30.36 | -7.67 | 3598 | 406.10 |
|  | Gramine | -32.77 | -9.83 | 3258 | 341.10 |
|  | Estragole | -30.54 | -8.98 | 2906 | 377.00 |
|  | Helenalin | -40.88 | -11.02 | 3520 | 387.20 |
|  | Glaucarubinone | -39.11 | -8.61 | 4816 | 569.80 |
|  | Limonene | -26.62 | -7.92 | 3040 | 320.10 |
|  | Linalool | -26.22 | -8.16 | 3372 | 389.50 |
|  | Jervine | -40.31 | -9.89 | 4844 | 564.30 |
|  | Menthone | -27.08 | -7.59 | 2958 | 316.40 |
|  | Myosmine | -30.37 | -8.71 | 2934 | 313.90 |
|  | Methylchavicol | -43.76 | -13.17 | 4276 | 457.80 |
|  | Mesaconitine | -38.22 | -5.25 | 5712 | 722.80 |
|  | Myristicin | -29.08 | -8.26 | 3306 | 351.40 |
|  | Picrotoxinin | -41.49 | -12.01 | 3394 | 373.20 |
|  | Piperine | -37.90 | -10.36 | 4190 | 536.80 |
|  | Neriifolin | -37.16 | -14.32 | 5402 | 747.20 |
|  | Piperitenone Oxide | -28.55 | -7.71 | 3018 | 325.90 |
|  | Plumbagin | -29.32 | -8.82 | 3086 | 372.80 |
|  | Psoralen | -33.71 | -9.13 | 3112 | 332.80 |
|  | Pulegone | -29.55 | -7.85 | 3000 | 317.40 |
|  | Podophyllotoxin | -47.54 | -13.71 | 4718 | 606.80 |
|  | Ricinine | -24.90 | -8.05 | 2808 | 346.80 |
|  | Sesamin | -35.61 | -7.32 | 4304 | 490.20 |
|  | Thymol | -30.18 | -9.15 | 2904 | 375.70 |
|  | Rotenone | -45.24 | -16.60 | 5088 | 642.20 |
|  | Napelline | -36.81 | -11.95 | 4342 | 519.60 |
|  | Physostigmine | -40.85 | -11.42 | 3954 | 464.30 |
|  | A Terpineol | -26.84 | -7.91 | 3000 | 321.70 |
|  | Caryophyllene Oxide | -35.41 | -9.35 | 3624 | 382.90 |
|  | Isopimpinellin | -32.40 | -10.38 | 3522 | 459.40 |
|  | Nicotine | -33.08 | -10.16 | 2982 | 319.40 |
|  | Sparteine | -38.79 | -12.00 | 3494 | 371.70 |
|  | Himachalol | -36.96 | -10.15 | 3380 | 361.90 |
|  | Asarinin | -38.36 | -9.16 | 4300 | 484.80 |
|  | Malathion | -26.25 | -6.60 | 3946 | 446.40 |

| ***Macromolecules*** | ***Ligands/ Metabolites*** | ***Global energy***  ***(Kcal/mol)*** | ***ACE*** | ***Score*** | ***Area*** |
| --- | --- | --- | --- | --- | --- |
| ***ABCB6 Protein*** | A Pinene | -25.38 | -7.12 | 3372 | 359.80 |
|  | A Terpineol | -24.17 | -5.73 | 3296 | 372.40 |
|  | A Thujone | -25.63 | -6.44 | 3354 | 355.30 |
|  | Anabasine | -25.38 | -7.80 | 3374 | 378.10 |
|  | Anethole | -28.28 | -7.64 | 2848 | 370.20 |
|  | Aconitine | -45.98 | -11.59 | 5670 | 738.80 |
|  | Absinthin | -37.41 | -6.74 | 5086 | 597.90 |
|  | Aphylline | -33.80 | -9.67 | 3604 | 442.10 |
|  | Anonaine | -35.52 | -10.74 | 3800 | 539.10 |
|  | Ascaridol | -26.63 | -7.56 | 3254 | 372.40 |
|  | Apiole | -26.76 | -5.88 | 3534 | 469.10 |
|  | Asarinin | -35.31 | -7.97 | 4190 | 502.70 |
|  | B Asarone | -29.66 | -7.34 | 3916 | 458.30 |
|  | Bergapten | -29.02 | -5.78 | 3436 | 395.60 |
|  | Caffeine | -24.08 | -5.79 | 3216 | 367.20 |
|  | Camptothecin | -36.00 | -9.44 | 4418 | 545.20 |
|  | Caryophyllene Oxide | -35.89 | -9.14 | 3470 | 443.10 |
|  | Cinerins | -38.86 | -11.94 | 4512 | 527.30 |
|  | Carvone | -25.16 | -7.08 | 2876 | 372.40 |
|  | Cinnamaldehyde | -25.70 | -7.49 | 2862 | 335.40 |
|  | Celabenzine | -37.97 | -10.93 | 5032 | 549.10 |
|  | Deguelin | -39.97 | -11.18 | 4894 | 641.20 |
|  | Delcosine | -36.59 | -7.70 | 4568 | 547.40 |
|  | Dillapiol | -29.74 | -7.53 | 3384 | 434.00 |
|  | Estragole | -24.16 | -6.13 | 3224 | 375.00 |
|  | Delcoline | -40.85 | -10.09 | 4882 | 606.60 |
|  | Elemicin | -24.58 | -5.60 | 3616 | 444.00 |
|  | Gedunine | -25.27 | -5.81 | 3360 | 382.30 |
|  | Galanthamine | -33.24 | -8.43 | 3874 | 473.60 |
|  | Gramine | -27.16 | -7.39 | 3454 | 385.00 |
|  | Helenalin | -27.84 | -7.56 | 3602 | 474.70 |
|  | Himachalol | -30.55 | -7.18 | 3428 | 446.50 |
|  | Glaucarubinone | -35.84 | -7.97 | 5022 | 583.90 |
|  | Isopimpinellin | -27.98 | -8.36 | 3296 | 362.60 |
|  | Limonine | -21.61 | -4.78 | 3110 | 341.90 |
|  | Menthone | -24.32 | -6.46 | 3176 | 367.20 |
|  | Jervine | -43.92 | -12.52 | 5280 | 616.50 |
|  | Myristicin | -28.34 | -7.11 | 3238 | 389.00 |
|  | Mesaconitine | -41.95 | -6.41 | 5670 | 702.90 |
|  | Necotine | -27.92 | -7.26 | 3568 | 380.50 |
|  | Napelline | -40.38 | -10.31 | 4462 | 527.30 |
|  | Myosmine | -29.54 | -9.07 | 2972 | 359.60 |
|  | Methyl Chavicol | -38.38 | -9.73 | 4118 | 603.50 |
|  | Neriifolin | -43.89 | -9.03 | 6712 | 756.20 |
|  | Picrotoxinin | -33.68 | -9.19 | 3398 | 377.30 |
|  | Physostigmine | -32.87 | -8.23 | 4198 | 489.30 |
|  | Piperitenone Oxide | -24.88 | -5.95 | 3370 | 360.50 |
|  | Plumbagin | -27.14 | -6.96 | 3114 | 361.20 |
|  | Psoralen | -27.88 | -8.35 | 3072 | 378.90 |
|  | Pulegone | -23.13 | -5.82 | 3218 | 357.70 |
|  | Ricinine | -29.09 | -9.35 | 2986 | 351.40 |
|  | Podophyllotoxin | -37.89 | -9.37 | 4598 | 657.90 |
|  | Sparteine | -41.39 | -9.86 | 3624 | 435.00 |
|  | Thymol | -24.94 | -5.87 | 3408 | 375.00 |
|  | Sesamin | -42.21 | -10.75 | *5162* | 654.20 |
|  | Rotenone | -41.91 | -8.73 | 5180 | 694.90 |
|  | Piperine | -44.24 | -12.71 | 4782 | 614.40 |
|  | Linalool | -23.39 | -5.47 | 3544 | 385.60 |
|  | Ephedrine | -24.45 | -5.93 | 3304 | 378.50 |
|  | Malathion | -28.17 | -6.74 | 4058 | 519.30 |
